# Awareness and Approaches Regarding Artificial Intelligence in Dentistry: A Scoping Review

**DOI:** 10.7759/cureus.51825

**Published:** 2024-01-07

**Authors:** Sultan Aldakhil, Khalid Alkhurayji, Shug Albarrak, Abdulaziz Almihbash, Rayan Aldalan, Khaled Alshehri, Salman Alrusaini, Ali Asiri

**Affiliations:** 1 Restorative & Prosthetic Dental Sciences, College of Dentistry, King Saud bin Abdulaziz University for Health Sciences, Riyadh, SAU; 2 Dentistry, King Abdullah International Medical Research Center, Ministry of National Guard Health Affairs, Riyadh, SAU; 3 Oral and Dental Health, Dental Center, Prince Sultan Military Medical City, Riyadh, SAU; 4 Health Information Management and Technology, College of Public Health, Imam Abdulrahman Bin Faisal University, Riyadh, SAU; 5 Restorative and Prosthetic Dental Sciences, College of Dentistry, King Saud bin Abdulaziz University for Health Sciences, Riyadh, SAU; 6 Maxillofacial Surgery and Diagnostic Sciences, College of Dentistry, King Saud bin Abdulaziz University for Health Sciences, Riyadh, SAU; 7 Dentistry, College of Dentistry, King Saud bin Abdulaziz University for Health Sciences, Riyadh, SAU; 8 Dentistry, College of Dentistry, King Saud bin Abdulaziz University for Health Sciences, Riaydh, SAU; 9 Dental Public Health, Epidemiology, and Public Health, Faculty of Population Health Sciences, Institute of Epidemiology and Health Care, University College London, London, GBR

**Keywords:** deep learning artificial intelligence, knowledge, ai and machine learning, dentistry, attitude, perception, awareness, artifical intelligence

## Abstract

Background: Dentistry is one of the unique specialties that deals with both humans and machines. This fact illustrates the strong potential for artificial intelligence (AI) implementation in dentistry, which makes awareness and attitude toward AI an important indicator for the future of this technology in the field. Hence, this scoping review aimed to report the status of awareness and attitude toward AI in dentistry.

Methodology: To ensure the quality and transparency of the present review, the Preferred Reporting Items for Systematic Reviews and Meta-analysis (PRISMA) flow chart is reported. Four databases were searched for related topics (Medical Literature Analysis and Retrieval System Online (MEDLINE), Excerpta Medica database (EMBASE), Google Scholar, and Scopus); 1,430 studies were identified, and after screening and filtering, 21 cross-sectional studies were included.

Results: Twenty-one cross-sectional studies were included and yielded 7,688 participants. With an average level of 50.31% among all the studies that reported awareness (18 studies). Four subgroups’ average levels of awareness toward AI in dentistry were reported: 67.16% among dentists, 42.58% among dental students, 45.56% for studies conducted on both dentists and dental students, and 69.53% for studies reporting awareness of AI in oral radiology. Regarding attitude, out of 13 studies, an average level of 44.13% felt threatened or thought AI would replace them.

Conclusion: The average level of awareness is in accordance with the attitude toward AI in dentistry. The low levels of awareness are important indicators of the gap formed between the inevitable application of AI and the lack of utilization in the dental field. AI implementation in dental schools’ curricula is required since the lowest reported level among subgroups was among dental students.

## Introduction and background

**Figure 1:**
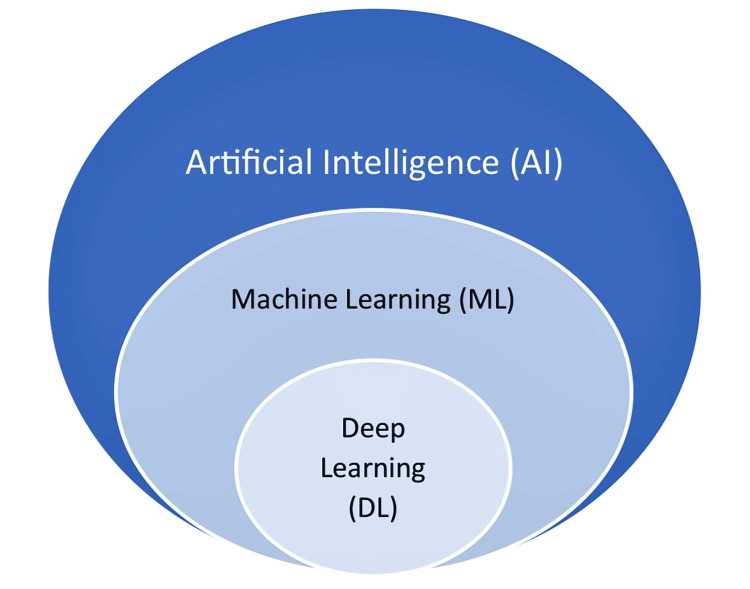
AI, ML, and DL integration The figure is the author's creation.

The last decade has introduced us to a relatively new technology that can change multiple aspects of dentistry as we know it today. Artificial intelligence (AI) is this new emerging technology with which the dental field has the ability to revolutionize the practice and help elevate the quality of the provided services [1]. Although AI is still a branch of computer science, it is designed to exhibit intelligent behaviors that can mimic human behavior, like understanding language, problem-solving, learning, reasoning, and many more [2]. Artificial intelligence is the big umbrella under which machine learning (ML) and deep learning (DL) fall (Figure 1).

Dentistry is one of the specialties that deals with both humans and machines, so the application of AI in the dental field is growing on a steady basis due to increased demand and the variety of possible applications in different dental specialties. Therefore, we can trace the up-to-date applications in the diagnosis of many conditions, including but not limited to vertical root fracture [3], dental caries [4], orthodontic cases [5], and oral and maxillofacial radiology [6]. Hence, the application of AI in dentistry is inevitable, and the practice of dentistry is going to evolve to adapt to this new era of science [7]. The movement toward AI seems to be a pleasure to some clinicians and a priority to others; however, the change is happening rapidly, and the need to be updated in this field does not seem to be a pleasure anymore. The solutions provided by AI are increasingly utilized by doctors, since their decisions are positively impacted by the augmentation of AI in various aspects of healthcare, including therapeutic protocols, diagnostic suggestions, personalized medicine, patient monitoring, and predicting and tracking epidemiological diseases [8].

Awareness, knowledge, and attitudes regarding AI in dentistry are areas that need further investigation to link this information with the increasing application of AI in the field. The studies that discussed this subject are rising in numbers, and the correlation of this knowledge with the practice of AI in dentistry can provide the full picture in which any gap in between can be successfully filled to obtain the best outcome of using this technology in dentistry. Many studies on this subject have reported that dentistry is one of the most important fields that AI has been incorporated into; however, the high levels of awareness of AI are not in accordance with the low levels of utilization of AI applications in dentistry [9]. Consequently, some articles reported the concern of some dentists that AI is a threat to them in the future [10], while other articles reported that not all dentists are threatened or afraid to be replaced by AI in the near future [11]. This review article aims to understand the level of awareness, perceptions, and attitudes toward AI in dentistry to help provide a basis on which AI can be implemented more effectively in dentistry to minimize the gap between awareness and utilization.

## Review

Methodology

**Figure 2:**
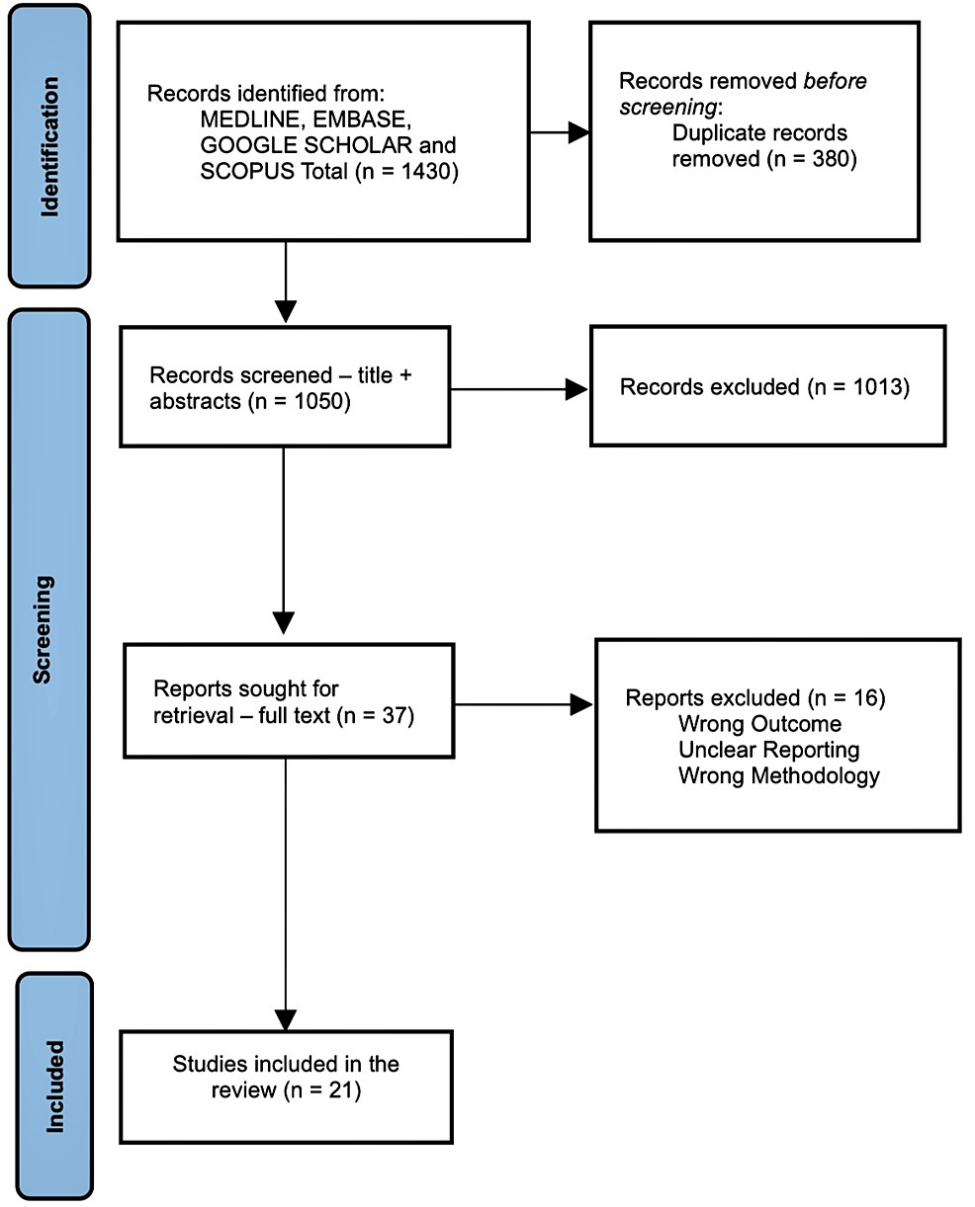
Preferred Reporting Items for Systematic Reviews and Meta-analysis (PRISMA) flowchart outlining the study selection process MEDLINE: Medical Literature Analysis and Retrieval System Online; Embase: Excerpta Medica database

This review article was undertaken as a scoping review since this type of review is suitable for emerging topics. To ensure the quality and transparency of the present review, the Preferred Reporting Items for Systematic Reviews and Meta-analysis (PRISMA) guidelines were used. The flowchart outlining the study selection process is presented in Figure 2.

Four databases were searched for related topics: Medical Literature Analysis and Retrieval System Online (MEDLINE), Excerpta Medica Database (EMBASE), Google Scholar, and Scopus. Each database was searched accordingly for the keywords namely, "awareness", "perception", "knowledge", "attitude", "artificial intelligence", "machine learning", and "deep learning"). Articles that discussed ML and DL were included as well since both models are still parts of AI (Figure 1).

Inclusion criteria were any articles that discussed the awareness, attitudes, knowledge, and perceptions regarding AI, ML, and DL in dentistry, while exclusion criteria were any articles that discussed these subjects in any field other than dentistry (for instance, studies that were done in the medical field). Three reviewers were included in the filtering and screening (S.A., K.K., and A.A.). In accordance with the scoping review guidelines provided by PRISMA, no quality assessment was performed.

Results

After the successful application of the inclusion and exclusion criteria, 21 cross-sectional studies were included, and each study was broken down based on article title, year of publication, study type, population, awareness levels, and attitudes toward AI (Table 1).

**Table 1:** Characteristics of the included studies *Articles about AI application in oral radiology; **Awareness of the differences between AI and robotics AI: artificial intelligence

Article	Year of publication	Country	Population (number)	Level of awareness/ knowledge	Attitude toward AI
Yüzbaşıoğlu et al. [11]	2020	Turkey	Dental students (1103)	48.40%	28.6% agreed that dentists will be substituted by AI.
Sur et al. [12]*	2020	India	Dentists (250)	68%	51% agreed that the major function of AI will be only the interpretation of complicated radiographic scans.
Sajjad et al. [9]	2021	Karachi, Pakistan	Dentists (118)	70.3%	All (100%) of the participants agreed that AI should be a part of dental treatment.
Soujanya et al. [13]	2021	Northern Telangana, India	Dentists and dental students (24)	62%	45.83% viewed that AI will not replace the role of dentist.
Pauwels et al. [14]*	2021	Brazil	Dentists and dental students (293)	37%	33.5% agreed that AI will replace oral radiologists in the next 15 years.
Khanagar et al. [15]	2021	Riyadh, Saudi Arabia	Dental students (423)	44.2%	39.7% reported that AI will lead to major advances in dentistry.
Keser and Pekiner et al. [16]*	2021	Turkey	Dental students (140)	60%	41.1% of the participants were not sure if AI would make a better diagnosis than a human doctor.
Abouzeid et al. [17]	2021	Saudi Arabia	Dentists and dental students (570)	7%**	40.3% did not agree that AI will replace dentists.
Jethlia et al. [18]	2022	Saudi Arabia	Dentists (200)	64.5%	44% agreed that the diagnostic abilities of AI are better than the clinical experience of a doctor.
Akthar et al. [19]	2022	Karachi, Pakistan	Dental students (355)	58.3%	31.5% responded that AI could replace them in the future.
Thulasi et al [20]	2022	North Gujarat, India	Dentists and dental students (200)	NA	53.5% of the participants thought that AI cannot replace the role of dentists.
Vamshi Ram et al. [21]	2022	Jammu & Kashmir, India	Dental students (100)	84%	N/A
Özel et al. [22]	2022	Turkey	Dental students (236)	23.73%	80% were not worried that AI will replace dentists.
Aboalshamat [23]	2022	Saudi Arabia	Dentists and dental students (389)	42.2%	49.1% agreed or strongly agreed that AI could replace dentists.
Singh et al. [24]	2023	India	Dentists and dental students (937)	51.3%	N/A
Karan-Romero M et al. [25]	2023	Peru	Dental students (200)	NA	45% disagreed that AI will replace dentists.
Hamd et al. [26]	2023	United Arab Emirates	Dentists and dental students (134)	42.5%	31.3% reported that AI would threaten/disrupt their profession.
Sridhar et al. [27]*	2023	India	Dentists and dental students (460)	94.13%	76.52% feared that AI will replace clinicians.
Kalaimani et al. [28]	2023	India	Dentists and dental students (1,000)	63.5%	26.9% agreed that AI will replace the role of dentists.
Rao et al. [10]	2023	India	Dental teaching faculty (407)	67.6%	93.9% believed that AI would be a threat to dentists in the future.
Elhijazi et al. [29]	2023	Morocco	Dental students (149)	16.8%	77.78% of the participants were confident in the use of AI in dentistry.

Regarding the year of publication, two studies were conducted in 2020, six studies were conducted in 2021, six studies were conducted in 2022, and seven studies were conducted in 2023. Eight studies were conducted on dental students, four studies were conducted on dentists, and nine studies were conducted on both dentists and dental students. Regarding the geographical distribution of the studies, it was as follows: India (eight studies), Saudi Arabia (four studies), Turkey (three studies), Pakistan (two studies), Morroco (one study), Peru (one study), Brazil (one study), and the United Arab Emirates (one study). The term "dentist" includes both clinicians and academicians of any level or position.

The total number of participants in the included studies is 7,688. Four subgroups’ average levels of awareness toward AI in dentistry were reported: 67.16% for studies done on dentists, 42.58% for studies done on dental students, 45.56% for studies done on both dentists and dental students, and 69.53% for studies reporting awareness of AI in oral radiology. Eighteen studies reported awareness, with an average level of awareness of 50.31%. Regarding attitude, out of 13 studies, an average level of 44.13% felt threatened or thought AI would replace them. Awareness towards AI application in dentistry is mentioned in Table 1. Awareness towards AI in general is also mentioned.

Studies Conducted on Dental Students

Awareness and knowledge: Yüzbaşıoğlu et al. [11], with a total number of participants of 1,103, reported that only less than half of the study’s participants (48.40%) had basic knowledge of AI. However, the study’s participants were willing to improve their knowledge in this field. Khanager et al. [15] reported that only 44.2% of the 423 participants were aware of AI applications in dentistry. Keser and Pekiner et al. [16] reported the awareness of oral radiology and AI to be 60% among 140 participants. Akthar et al. [19] reported 58.3% awareness among 355 participants. Vamshi Ram et al. [21] reported that 84% of the 100 participants were aware of AI applications in dentistry. Özel et al. [22] reported the lowest level of awareness (23.73%) among 236 participants. Elhijazi et al. [29] reported only 16.8% awareness out of 149 participants toward the application of AI in dentistry.

Attitude: Yüzbaşıoğlu et al. [11] reported that only 28.6% of the study participants (1,103) agreed that AI could replace them in the near future. Khanager et al. [15] reported that 39.7% of the participants thought that AI would lead to major advances in dentistry. Keser and Pekiner et al. [16] reported that 41.1% of the participants were not sure if AI would make a better diagnosis than a human doctor. Akthar et al. [19] reported that 31.5% of the participants responded that AI could replace them in the future. Özel et al. [22] reported the highest number in the included articles in the number of participants who were not worried that AI would replace them (80%). Karan-Romero et al. [25] reported that out of the 200 study participants, 45% disagreed that AI would replace dentists. Elhijazi et al. [29] reported that 77.78% of the participants were confident in the use of AI in dentistry.

Studies Conducted on Dentists

Awareness and knowledge: Sajjad et al. [9] reported that out of 118 participants, the awareness level was 70.3%. According to Rao et al. [10], the reported awareness rate was 67.7% out of 407 participants, which was relatively high. Sur et al. [12] reported that out of 250 participants, the awareness level was 68%. Regarding the attitude, 51% agreed that the major function of AI will be the interpretation of complicated radiographic scans. Jethlia et al. [18] reported 64.5% awareness among the 200 participants.

Attitude: Sajjad et al. [9] reported that all (100%) of the participants agreed that AI should be a part of dental treatment. Rao et al. [10] reported that a high number of the participants (93.9%) believed that AI is a threat to dentists. Sur et al. [12] reported that 51% agreed that a major function of AI would be the interpretation of complicated radiographic scans. Jethlia et al. [18] reported that 44% agreed that the diagnostic ability of AI is better than the clinical experience of a doctor.

Studies Conducted on Dentists and Dental Students

Awareness and knowledge: Soujanya et al. [13] reported a relatively high level of awareness (62%) among the 24 study participants. Pauwels et al. [14] reported awareness of AI in radiology but not in dentistry in general. The awareness level was 37% among the 293 study participants. Abouzeid et al. [17] did not report the awareness of AI; however, they reported the awareness of the differences between AI and robotics (7%), and as a result, it was not included in the mean number of awareness. Aboalshamat [23] reported that of the 389 participants, less than half (42.2%) reported that they know AI uses in dentistry. Almost the same number (42.5) was reported by Hamd et al. [26], while the number of study participants was lower (134). Singh et al. [24] reported 51.3% awareness among the 937 study participants. Sridhar et al. [27] reported a 94.13% level of awareness among 460 participants. Kalaimani et al. [28] reported a 63.5% level of awareness among 1000 participants.

Attitude: Soujanya et al. [13] reported that 45.83% of 24 participants viewed the role of dentists as not being replaced by AI. Pauwels et al. [14] reported that 33.5% of 293 participants agreed that AI will replace oral radiologists in the next 15 years. Abouzeid et al. [15] reported that 40.3% of 570 participants did not agree that AI would replace dentists. Aboalshamat [23] reported that among the study participants (389), almost half (49.1%) agreed or strongly agreed that AI could replace dentists. Hamd et al. [26] reported that 31.3% of the 134 study’s participants reported that AI would threaten or disrupt their profession. Sridhar et al. [27] reported that 76.52% feared that AI was going to replace clinicians. Kalaimani et al. [28] reported that 26.9% agreed that AI would replace the role of dentists.

Discussion

Studies Conducted on AI in Dentistry

Awareness: The present review successfully reported 21 cross-sectional studies about awareness and attitude toward AI in dentistry. Out of the 21 reported articles, 18 studies reported awareness toward AI, with an average level of awareness of 50.31%. Surprisingly, the lowest level of awareness among dental students (16.80%) toward AI was reported by Elhijazi et al. [29] in 2023, while the highest level of awareness (80%) was reported by Vamshi Ram et al. [21] in 2022. Since the most recent reported article has the lowest level of awareness, this can be attributed to the undetermined efforts in the field to raise awareness. However, other factors, like the geographic variation between the studies, can cause uncertainty among the different levels of awareness; one study was in India and the other was done in Morocco. Elhijazi et al. [29] have taken a further step in assessing awareness, where the students were provided with a short course about AI to reassess their awareness and willingness toward AI learning. The level of knowledge of AI was 16.8%; in comparison, after the course, a level of 62.5% knowledge of the basis of AI operations in dentistry was reported. These results revealed insufficient knowledge about AI, and correlatedly, an optimistic positive attitude toward the technology is reported.

Regarding the studies done on dentists, 70.03% awareness was the highest reported level by Sajjad et al. [9] in 2021, while 64.5% was the lowest reported level by Jethlia et al. [18] in 2022. Despite the geographic variation between both studies, another factor related to the study’s population was reported. As in the first study [9], consultants, specialists, and postgraduate trainees were included without any inclusion of dental interns, while the other article [18] included dental interns, which may have led to this discrepancy in results. In the study by Jethlia et al. [18], the level of awareness toward AI, in general, was 74%, which was higher than awareness of AI in the dental field, considering that the study participants were from the field of dentistry and were dental interns and dental practitioners; a fact that further emphasized the importance of knowledge of AI’s concepts and the obvious gap in dental education on this subject. Although the levels of awareness in studies reporting dentists’ awareness toward AI in dentistry are considered to be high among the studies reported in this review, these levels have the potential to be increased due to the willingness of the participants to learn more about AI and to adopt its technology. By the same token, 85.5% of Jethlia et al. [18] believed that AI would be valuable in dentistry.

The average level of awareness among dentists was 67.16%, which was higher than that of dental students, which was 42.58%. This raises the issue of the lack of implementation of AI in the dental schools’ curricula and makes AI, to date, just a supplemental science that is not yet taught thoroughly in dental schools. Khanagar et al. [15] reported that although the dental students’ awareness was considered low, the students appreciated the changes happening with AI and would try to learn more about its applications. This information clarifies the major role of dental schools and dental institutions in putting greater emphasis on delivering and creating initiatives that can withstand the huge impact of AI and its developments in dentistry. The judgment cannot be made until serious actions toward this issue are taken to counteract the low levels of awareness, which do not come in accordance with the reported enthusiasm to learn more about the technology.

Regarding studies that were done on both dentists and dental students, the highest level of awareness was reported at 62% in 2021 by Soujanya et al. [13], while the lowest level was reported at 42.2% in 2022 by Aboalshamat [23]. The highest level reported by the first study may be attributed to the low number of participants (24) in comparison to the lowest reported number, which was among 389 participants. To overcome this variation, the average level of awareness was calculated with respect to the total number of participants in the nine included studies, yielding an average level of 45.56%. This average level is close to the average level of the 18 studies (50.31%) that reported awareness of AI, which assures the consistency of the reported levels of awareness among individuals in the dental field. These levels are still considered to fall short of the developments being initiated and the unavoidable implementation of AI in dentistry.

Attitude: The attitude toward AI is not consistent among the reported studies; however, the average level of participants who felt threatened or thought AI would replace them in the future was 44.13% reported from the 13 included studies. The highest level of participants who believed that AI could replace dentists was 49.1%, which was reported by Aboalshamat [23]. In contrast, Özel et al. [22] reported that 80% of the study participants were not worried that AI would replace dentists. Rao et al. [10] reported the highest percentage (93.9%) of participants who believed that AI was a threat to them. This high number is believed to affect the willingness to embrace AI technology negatively. Other studies, like the one by Thulasi et al. [20], also reported a moderate number of participants (50.35%) who believed that AI could not replace them. Similarly, Karan-Romero et al. [25] reported that 45% of the study’s participants disagreed that AI would replace dentists.

The fear of AI is a well-reported fact in this review; however, optimistic views and enthusiasm toward understanding and adopting this technology are also reported. Elhijazi et al. [29] reported an effective example of a positive attitude toward AI where the study participants were exposed to a course on AI in dentistry, and their knowledge increased dramatically after the course, which indicated a willingness to know more about the technology. This can clearly provide a vision of a positive attitude toward AI, especially when it is taught well and the myths and misconceptions are eliminated. No correlation was found between the level of awareness and the attitude toward AI. For instance, Sajjad et al. [9] reported the highest level of awareness among studies done on dentists and 100% agreed that AI should be a part of dental treatment. While Rao et al. [10] reported a level of awareness (67.6%) that was close to the previous level, 93.9% of participants reported that AI would replace them. However, these variations could be due to the personal beliefs of the study’s participants and their different exposures to myths and misconceptions about AI. A further explanation that was offered by Bewersdorff et al. [30] is that the view of AI is binary, where it is viewed to be dangerous and beneficial simultaneously. The interpretation of the included studies seemed to be in accordance with this explanation, as the views of AI differ and the attitude does not have a consistent pattern based on which it can be reported.

Studies Conducted on AI Awareness and Attitude in Oral Radiology

Regarding the studies that reported the level of awareness and attitude towards AI in oral radiology, the average level of awareness was 69.53% among the four studies. This may be attributed to the easy manipulation of AI applications by dentists in the field of oral radiology. However, this awareness is still linked with a level of anxiety, as Pauwels et al. [14] reported that 35.5% of the study participants believed that AI would replace oral radiologists as early as 15 years, and Sridhar et al. [27] reported that 76.52% believed that AI would replace dentists. In contrast, Sur et al. [12] reported that more than half of the study’s participants believed that the major function of AI was going to be limited to the interpretation of complicated radiographs, while Keser and Pekiner et al. [16] reported that 41.1% of the study’s participants were not sure if AI was going to provide a better interpretation than a human doctor. The high dependency in the field of oral radiology on interpretation is going to increase the demand for AI applications, each of which will try to deliver the best outcomes to eliminate the need for oral radiologists. While the need for oral radiologists is not linked only with interpretation, AI does not seem to be close to replacing oral radiologists, at least in the near future, since the need for oral radiologists who can understand the characteristics of the images is highly important even for further development of AI applications [31].

Although AI is not a new concept, the last decade has witnessed an increase in demand and application of AI concepts in multiple sciences. This increase was accompanied by different theories about AI and how the population dealt with its presence and effect on the job market worldwide. Bewersdorff et al. [30] in 2023 aimed to assess the myths and preconceptions or misconceptions of artificial intelligence and concluded that there are a variety of myths and preconceptions or misconceptions of AI. For instance, there is a huge misconception that AI is only for computer scientists and people in the programming industry. This can be related to dentistry: people in the dental field do not have a solid background in computer science, so anything related to AI is exposed to a shortsighted view, which may affect their attitude and adoption. Such misconceptions put people in a constant state of fear towards AI, which affects their perception of AI developments. It concluded that AI should be implemented more in school courses and curricula. Implementation of AI in educational systems will provide the students with a deeper understanding of the developments and, hence, make the utilization of its features easier.

The limitation of the present review is the unequal geographical distribution of the included studies. The reported studies were founded on three continents only (Asia, Africa, and South America). This fact affects the generalizability of the review as there are some differences in social, economic, and academic infrastructure in comparison to other continents and countries. Further investigations and articles are required to identify the key points that can change the perception and perspective of the dental field toward AI. Moreover, the attitude toward AI is still not clearly reported. Assuming that clarity is linked to knowledge, more studies should be conducted on individuals who have high levels of knowledge about AI to clearly report their attitude toward this technology.

## Conclusions

The levels of awareness toward AI in dentistry are still not satisfying and are considered to be low since almost only half of the included studies’ participants were aware of AI. The highest level of awareness was reported among studies conducted among dentists only, while the lowest level of awareness was reported among studies conducted on dental students. This variation can be linked to the lack of implementation of AI in dental schools’ courses and curricula.

The eagerness to learn more about AI applications and developments by almost all the participants of the studies is not in correlation with the undetermined efforts being made by dental institutions and schools. It is important to take serious action to accommodate this enthusiasm. Further concentration is needed to keep up with the inevitable developments of AI in dentistry. This will enable the new generation of dentists to lead the developments in AI and not resist them, as almost less than half of the participants in the included studies felt threatened or believed that AI would replace them.
